# Understanding the impact of COVID-19 on youth sport in Australia and consequences for future participation and retention

**DOI:** 10.1186/s12889-021-10505-5

**Published:** 2021-03-05

**Authors:** Sam Elliott, M. J. Drummond, I. Prichard, R. Eime, C. Drummond, R. Mason

**Affiliations:** 1 SHAPE Research Centre, Flinders University, Adelaide, South Australia; 2 School of Science, Psychology and Sport, Federation University, Ballarat, Victoria Australia; 3 Institute for Health and Sport, Victoria University, Melbourne, Victoria Australia; 4 Melbourne Graduate School of Education, University of Melbourne, Melbourne, Victoria Australia

**Keywords:** Youth sport, Participation, Pandemic, Retention, Coronavirus, COVID-19

## Abstract

**Background:**

COVID-19 continues to represent the single biggest challenge to contemporary community sport globally. Compliance with social distancing policies, strict return-to-play protocols, and COVID-19 specific training has, perhaps, forever changed the way that children and young people engage in organised sport. Within this context, and while many children and families seek to re-engage with community sport, we (researchers and sport practitioners) have an obligation to ask questions about how the pandemic has impacted youth sport, understand the short- and long-term consequences, and explore what (if any) opportunities can be seized to assist and improve future participation and retention. The aim of this paper was to present an in-depth exploration of the impact of COVID-19 on youth sport in South Australia.

**Methods:**

Within an interpretive descriptive methodology, this qualitative investigation draws on rich, individual interview and focus group data with 39 youth (ages 15–18), parents, coaches, and sport administrators. A reflexive thematic analysis was undertaken, leading to the development of four substantive themes.

**Results:**

We conceptualised the ‘4 Rs’ to advance theoretical understandings about the pandemic’s impact on youth sport, including the themes ‘recognising struggle’, ‘reconnection’, ‘re-engaging after restrictions, and ‘reimagining sport’. The themes captured insights about a decline in mental wellbeing and physical activity, an increase in family connectedness, the challenge for sports to attract volunteers and participants back into sport, and the opportunities to reset values and philosophies underpinning the provision of youth sport.

**Conclusion:**

The findings provide valuable insight into the youth sport setting as a result of the global pandemic and suggest that families, sporting clubs and sporting organisations require additional resources and tools (for example, support for parents to facilitate their children’s training at home during lockdown) to aid recovery efforts and to ensure the survival and prosperity of youth sport into the future.

**Supplementary Information:**

The online version contains supplementary material available at 10.1186/s12889-021-10505-5.

## Background

Coronavirus disease-2019 (COVID-19) was declared a global pandemic by the World Health Organisation on March 11, 2020. Some 12 months on (February 2021), it is now arguably the greatest global state of emergency since World War II. Among many facets of life, sport is one area that has been heavily impacted. This is significant given the role and importance of sport in the lives of individuals, families and communities globally. It is recognised in sport policy across various nations that community-level sport plays a vital role in promoting positive social, emotional, cultural, and economic outcomes [1–3]. Within the Australian context, community sport also plays a vital role from social, emotional, cultural, and economic perspectives. As the pandemic continues to develop, nations including Australia are developing a series of measures to support youth sport participants’ ‘return-to-play’ (c.f [4].), contingent on individuals, families and club personnel strict adherence to physical distancing guidelines, return-to-play protocols, and undertake specific COVID-19 volunteer and officer training for sport events [5].

In Australia at the time of developing this paper, states and territories are in various stages of ‘lockdown’ and social distancing restrictions due to a second ‘wave’ of COVID-19 cases. States such as South Australia, which to date (03/02/2021) have had a total of 597 cases since March 2020, 4 deaths, and 1 current active case (https://www.covid-19.sa.gov.au/home/dashboard), have been able to govern a return to youth sport competition. Other states, such as Victoria (28,824 cases since March 2020, 909 deaths, 54 active cases), are emerging from lockdown and easing restrictions, with a ‘roadmap’ for return to community sport recently implemented.

### Youth sport ‘pre-COVID-19’

Before the pandemic (‘Pre-COVID-19’), youth community-level sport participation was a key priority of the Australian Government’s National Sporting Plan [1]. Australian sport was estimated to provide a combined economic, health and educational benefit of $83 billion annually to the nation [6]. Youth sport participation was of priori importance given that sustained involvement has been positively associated with improved wellbeing, confidence, self-esteem, and fewer depressive symptoms among individual participants [7]. Internationally, youth sport settings have been recognised for their contribution to children’s positive youth development via optimal social climates, a focus on life skill development and positive developmental outcomes spanning social, personal, and physical domains [8]. Additionally, community sport settings are gaining momentum as an efficacious site for the delivery of mental health and wellbeing interventions [9]. Thus, the broad and expansive potential benefits of youth sport have been clearly recognised.

Notwithstanding the associated benefits of long-term participation in organised sport, the broader ‘Pre-COVID-19’ landscape of youth sport also included complex social and cultural issues that continued to challenge the sport experience. In terms of growing participation and retaining participants, community-level sporting clubs have struggled to maximise participant fun and enjoyment by focusing on community sport as a talent pool for servicing the elite pathway, emphasising a focus on winning games [10]. This perspective corroborates other comprehensive reviews in which a lack of participatory fun has been identified as a primary determinant of sport dropout among children (for review, see [11]).

In addition to dropout, marginalised groups continued to encounter restrictive barriers to sport participation [12]. Organised sports often comprise exclusionary sites for the LGBTIQ+ community [13], individuals with disability [14], and ethnically diverse groups and migrant participants [15, 16], leading to increases in anxiety, dissatisfaction, and/or self-exclusion. Youth sport settings can also maintain forms of disadvantage for marginalised populations, especially if individual experiences of the broader sports system reinforces hegemonic power and ideals which work to disillusion, disengage and damage young people [17]. Furthermore, girls and young females continue to encounter a range of social, cultural and structural barriers to long-term sport participation including a lack of confidence in physical skill, the absence of legitimate and well developed pathway opportunities [18], undermining wider perceptions of progress and enhanced opportunities for youth sport participation.

Other pervasive issues surrounded parenting behaviours at children’s sporting events [19, 20]. In the Australian youth sport context, parents have been found to exert a largely positively influence on children’s enjoyment and motivation but they equally possess the potential to exacerbate feelings of anxiety after competition via post-game debriefs [21, 22]. Given that the function and survival of youth sport is contingent upon parents fulfilling core (e.g. coaching, umpiring, governance) and peripheral (e.g. social committee) voluntary roles [23], high levels of parental involvement in youth sport are critical. However, parental involvement can be challenging, especially when managing dual roles such as parent and coach, which has the potential to adversely impact participant enjoyment, motivation and overall engagement [24].

The complex ‘Pre-COVID-19’ Australian youth sporting landscape resonated with global perspectives about the state of contemporary youth sport (c.f [25].) and current priorities for the field (see [26]). For instance, in Gould’s [25] review of the youth sport landscape, he argued that further scientific attention was needed to address psychological (motivation, stress and burnout), structural (e.g. access, tracking participation and retention), cultural (e.g. professionalisation of youth sport), interpersonal (e.g. significant others), economic, government and legislative, and translational science issues. Similarly, Howie and colleagues [26] asserted that a focus on sport participation rates, physical activity from sport, the contribution of sport to health, and the overall return on investment from youth sport are complex but pertinent research priorities in order for the field to progress. Collectively, both Gould [25] and Howie et al. [26] articulate forceful commentaries about the universal challenges entrenched in the youth sport experience, providing important insights into the ‘Pre-COVID’ youth sport climate.

### The current context

While these practitioner- and researcher-focused priorities will most likely retain importance during and after the pandemic, the unexpected disruption to the youth sport context has forced scholars to additionally consider the impact of COVID-19 on youth sport both now and into the future. Since the beginning of the pandemic, several papers have critically forecast the challenges and considerations that need to be managed in order for elite athletes [27], disability sport participants [28], and sport managers [29], to resume involvement in organised sport once the impact of the pandemic recedes. Others have theorised pragmatic and research-focused consequences of the pandemic for youth sport. For instance, one of the major concerns proposed by Drummond et al. [30] is that the pandemic may lead to a potential generation of young people ‘lost’ to organised sport. While they acknowledge that many participants and volunteers will likely return to sport when it is safe to do so, they express concern about who *may not* return, and encourage researchers to ask ‘why?’ and ‘what can be done to help?’. Specifically, they argue that fee increases, a plausible reduction in parent and community volunteers, and a decrease in sport development officer roles are but a few examples of how the pandemic may represent a ‘tipping point’ for sporting clubs and families who find it difficult to return to sport.

Similar considerations were proposed by Kelly et al. [31] in their discussion paper about youth sport in the time of COVID-19. In an effort to ‘map out’ the far-reaching implications of COVID-19 on youth sport, they utilised the Personal Assets Framework [32] to consider the potential implications for youth sport development, and conceptual models such as the ‘4Cs’ (competence, confidence, connection and character) and the 3Ps (performance, participation and personal development) to describe the respective potential short- and long-term consequences for youth participants. Kelly and colleagues summarised their position with a series of contextual, methodological and practical considerations for researchers and practitioners, categorised into immediate, short-term and long-term outcomes. For instance, immediate considerations researchers and practitioners may seek to explore *how can parents, peers, coaches, and sport organizations effectively engage with each other both during the crisis and during return to play?* (contextual), *how political, sociocultural and geographical contexts might influence current sport experiences?* (methodological), and *what are the potential implications of children engaging in alternative activities during the pandemic on developmental outcomes?* (practical).

Beyond the theoretical ideas put forward by Drummond et al. [33], and the commentary by Kelly et al. [31], no empirical evidence exists to our knowledge about the impact of COVID-19 on youth sport. More specifically, and noting the question *How are youth sport stakeholders experiencing the effects of COVID-19 in real-time?* [31], rich accounts about the perceived impact of COVID-19 on youth sport are yet to be understood. Against this backdrop, the objective of the study presented in this paper was to explore the perceived impact of COVID-19 on youth sport stakeholders including youth (ages 15–18), parents, coaches and sport administrators) across South Australia. Two key research questions were the motivation driving the study:

1. What impact has the COVID-19 pandemic had on youth sport?
2. How has the COVID-19 pandemic influenced attitudes and behaviours regarding future youth sport participation?

## Methods

### Epistemology and study design

This study was underpinned by a relativist ontology and interpretivist epistemology, which posits that there are multiple realities rather than one single, objective truth (Sparkes & Smith, [34]). This paradigm is predicated on the ontological assumption that people actively construct and act upon realities they assign to events, actions, processes, ideologies, and conditions in the world [35]. Further, research rooted within an interpretivist epistemology focuses on how people make sense of their reality and how collective definitions of reality shape and direct human thoughts and behaviours [35]. In line with this approach, the ensuing means, methods and practices emphasised the collection of data from the viewpoint of the participant and the researcher’s attempt to interpret meanings, values, and explanations about an understudied phenomenon. In keeping with the epistemological underpinnings of the study, an interpretive descriptive research design was employed. Interpretive descriptive research designs are suitable for “why”, “how” and “what” questions about human behaviours, motives, thoughts, and perceptions and, thus, well suited for identifying and understanding a central phenomenon and/or developing theories, concepts, and frameworks [36] . Also known as ‘qualitative description’, an interpretive descriptive research approach is commonly adopted in studies seeking to generate insights and perspectives about how a group of people feel about, and experience, a phenomenon [36, 37].

In terms of positionality, the researchers were heterogenous in terms of race, age, and gender leading to both insider and outsider positions in relation to the research. Some researchers did not have any involvement in youth sport because they did not have children or were not involved as a coach or volunteer. In contrast, others were involved through their own children’s involvement in sport and/or through coaching youth teams. Subsequently, the researchers assumed an ‘insiders’ position in which there was some understanding about the nuances of the impact of the pandemic on youth sport, as well as an ‘outsiders’ position in which some preconceived ideas shaped their understandings about the phenomenon [38]. For instance, some assumptions about how the pandemic might influence children’s attitudes toward sport were declared by an ‘outsider’ researcher prior to commencing the project. This was part of a reflexive exercise in which the research team practiced being self-aware about their ideas, beliefs, motives, and presumptions with respect to the investigation. A series of means, methods and practices were used to promote reflexivity including the use of a critical friend (detailed later in the section *methodological rigor*) and incorporating a perspective taking technique [39] which involved utilising insider and outsider perspectives to generate a richer and more nuanced theoretical understanding of the phenomenon.

### Participants

Following institutional ethics approval from the University Social and Behavioural Research Ethics Committee (research project number 8647), 66 state sporting organisations were contacted via email to disseminate information about the study via their respective social media platforms. A total of 15 sporting organisations responded with intentions to assist the study’s recruitment efforts. The authors’ affiliated research centre also promoted recruitment information on their website, social media channels and via a mailing list of sport partners. The significance and timeliness of the project also garnered state-wide media attention. The authors (led by authors 1–2) received several opportunities to participate in online webinars, radio interviews and offer commentary for news stories which created additional opportunities to promote information about the study for potential participants. In total, 39 youth sport stakeholders (24 male, 15 female) were recruited as participants for the study. The participants represented a heterogeneous sample of youth athletes (15–17 years) (*n* = 18), parents (*n* = 11), coaches (*n* = 5), sport volunteers (*n* = 2), and sport administrators (*n* = 3) aged between 15 and 82 years from across the metropolitan, regional, rural, and remote areas of South Australia. In terms of ethnicity, the participants were predominately white, Anglo-Saxon except for two youth participants of Asian descent. No information was obtained on participants individual socioeconomic status. Youth athletes participated in a range of sports including Australian Rules football, soccer, netball, swimming, tennis, BMX bike racing, athletics and basketball. The parents and volunteers, who were not the parents of the youth participants in this study, were involved in Australian Rules football, water polo, netball, athletics, swimming, triathlons, and tennis. The coaches were involved in team sports including Australian Rules football and cricket. Finally, the administrators were involved in cricket, Australian Rules football and athletics. At the point of data collection, the level of sport involvement was predominately community- and state-level competition.

A broad geographical sample was achieved, in part, because social distancing restrictions at the time of data collection encouraged participants to use online videoconferencing platforms such as Zoom and Skype to participate in the study. Informed, written consent was gained from all participants, including written parental consent for participants aged under 18 years and written assent from youth participants aged under 18 years.

### Data collection

Online, individual interviews and focus groups were used for data collection. Individual interviews were used because they are the most common qualitative method of obtaining rich, descriptive qualitative data in the field of sport and exercise [40]. Similarly, focus groups were utilised for their capacity to invite dynamic dialogue and potential to proliferate different perspectives. Focus groups, consisting of groups of 3–4 youth participants, were organised by age (e.g. 15-year-old participants were grouped together) to promote group familiarity and comfort [41] while online interviews were conducted with adult stakeholders to support participation in convenient conditions and enable ease of access [42]. These considerations were important aspects of the overall research design given the impact of the pandemic on the lives of the participants.

Five focus group participants knew each other through competitive sport, while the others did not as they were involved in completely disparate sports and levels of competition. This had little impact on the overall focus group climate because all focus groups required significant effort on behalf of the researcher to foster a supportive and dynamic online climate. Some techniques involved using ice-breaking questions such as *To begin with and make sure our audio is working could I please ask for your name, your age and maybe what sport you play*?, and the use of screen sharing to visually display promoting materials such as social media stories about the pandemic and community sport. This gradually led to quite dynamic overall discussions, but several factors adversely impacted on the overall online experience. For instance, one challenge pertained to ‘spotty’ internet connectivity between the researcher and the participants which momentarily disrupted the ‘flow’ of conversation at times. A common consequence was that the researcher would attempt to reignite discussions while a participant continued to speak following a slight disruption to connectivity, creating an overlapping dialogue. Similarly, in one individual interview, the participant had to log in several times to complete the interview, expanding the interview duration considerably.

The individual interviews and focus groups were semi-structured, which involved the researchers using a pre-planned interview/discussion guide to ask the participant about relatively focused but open-ended questions about the impact of the COVID-19 pandemic on youth sport (see Additional File 1 and Additional File 2 to view individual interview and focus group guides). A semi-structured approach provided direction and flexibility for the participant and researcher to construct dialogue that invited storytelling, descriptions about their perspectives, insights and experiences, and reflective accounts about feeling, emotions and behaviours in relation to the research questions [43]. The added advantage of a semi-structured approach was that the researcher was able to ‘drill down’ and tease out more information with intuitive probing questions, based on the topics that were discussed and the issues that arose in the interview. Given the novel nature of the investigation, the main questions were developed from concerns and topics purported in the media about the impact of the pandemic on community and youth sport including coping strategies for dealing with the ‘lost’ sporting season and the role of parents in supporting alternative forms of sport and leisure time activity. The online, semi-structured individual interviews and focus groups ranged from 20 to 90 min in duration and produced 332 pages of 1.5 spaced textual data. Interviews were digitally recorded and transcribed verbatim using an independent contractor, third-party transcription service.

### Data analysis

NVivo software was used to assist the organisation of transcripts and support a recursive process of reflexive thematic analysis – and importantly, *not used* for the purpose of auto-coding. Reflexive thematic analysis was employed which involved a ‘backward and forth’ process of familiarisation with the data, coding, generating initial themes, reviewing themes, defining and naming themes and writing up [44]. The researchers (authors 1 and 6) read the transcripts for familiarity and began ‘sound boarding’ ideas that might be worthwhile exploring throughout the analysis. This was an important technique to ensure that the textual transcripts were verbatim accounts of the audio recordings, especially concerning the focus group data where multiple voices were captured. It was also necessary for enhancing familiarity with the data. Semantic coding was then undertaken (author 1 and 6) which involved labelling segments of text using a concept-by-concept (as opposed to line-by-line) method. A critical friend (authors 2–5) approach was employed at this point of analysis given that the phenomenon under investigation was not well understood and the researchers possessed varying degrees of experience coding qualitative data. The critical friends assumed insider (authors 2–3 and 5) and outsider (authors 4) positions to the research, which were deemed important in sound boarding initial ideas in the early stages of analysis (led by authors 1 and 6). This created a dynamic analysis in which multiple realities and coding interpretations were fully explored. It also provided a starting point for the researchers (authors 1 and 6) to commence the development of potential themes. Using NVivo, the relevant coded data extracts were collated within potential preliminary themes to ascertain if more robust themes could be constructed. The role of the critical friend technique was integral during this phase of the analysis (authors 2–5), reminding the researchers (authors 1 and 6) about the multiple ways in which the proposed ideas could be interpreted. A subsequent outcome was the review and optimisation of 13 potential candidate themes pertaining to the impact of the pandemic on youth sport. In reviewing the potential themes, several themes were aggregated together while others were abandoned owing to doubt about their analytical depth as standalone themes. As a result, four analytically cogent themes considered necessary for telling the overall ‘stories’ about the phenomenon were refined and named.

### Methodological rigor

We offer a novel combination of criteria in the form of a list for readers to judge the quality of the qualitative research presented within this paper. The basis for the ensuing combination is guided by the relativist ontology underpinning the study, which is not congruent with the adoption of ‘fixed’ universal criteria [45]. This was not a prescriptive list but rather the result of an open-ended approach to inquiry.

#### Worthy topic

Tracy [46] describes how research topics can emerge from disciplinary priorities but also note that ‘worthy’ topics ‘just as easily’ develop from timely societal and personal events. This research is unequivocally worthy given the far-reaching impact of the pandemic on nearly all aspects of society and culture. Although this research provides a timely response to academic recommendations [31] about understanding COVID-19 and its impact on youth sport, the research is clearly worthy insofar that it can be a conduit for participants stories, experiences, challenges and perceived opportunities to assist the construction of knowledge that can support sporting communities recovery when the impact of the pandemic recedes.

*Rich rigor.* To achieve rich rigor, thorough attention was given to sampling strategies that provided sufficient, abundant, and rich sources of qualitative data. The enactment of three recruitment strategies reflects the careful attention given to recruiting a sufficient sample (*N* = 39) that yielded 332 pages of textual data. Complexity, a key characteristic of rich rigor, was also maintained in the skilful facilitation of online individual interviews and focus groups. Specifically, where focus groups involving children (under 18 years) were used, complexity was maintained in early planning and scheduling, establishing a comfortable online ‘environment’, conducting the discussion, and working within an ethical framework to elicit mixed and varied responses from a variety of key stakeholders. Finally, in terms of interviewing, Tracy [46] asserts that rigor can be demonstrated by reporting the number and length of interviews, the types of questions asked, the level of transcription detail, the practices taken to ensure transcript accuracy, and the resultant number of pages of interview transcripts. We believe that these details have been provided in the methodology clearly for the reader to judge the rigor of the qualitative work undertaken.

#### Sincerity

Sincerity was practiced by adopting strategies that promoted reflexivity such as using a critical friend method during reflexive thematic analysis. However, the sincerity of the research is also marked by honesty and transparency about the successes and mistakes of the research evidenced in this paper. For instance, being open and honest about the difficulties of online qualitative data collection in this particular study amid the COVID-19 pandemic is but another example of practicing sincerity.

#### Credibility

The credibility of the research lies in the results, which are rich, thoughtful, and descriptive accounts to “show” rather than “tell” the readers about the phenomenon. The tools of persuasion to deliberately practice credible research include evocative and thick descriptions of the data and an immersive analysis that considers not only what is being said and by who, but also what is not being said. These methodological decisions are characterised throughout the research and especially in the analysis, which led to the development of an abundant, complex set of results in which multiple and varied voices are observed.

#### Resonance

While it will be up to the readers to judge the research against the criteria resonance, we consider the research has the capacity to meaningfully reverberate and affect an audience. Resonance is offered through the research and its potential to elicit a vicarious experience for readers as they connect the research with their own engagement in life’s affairs or tacit experiences. If rich theoretical expressions, contextual details and sufficient data bears “familial resemblances to the readers’ experiences, settings they move in, events they’ve observed or heard about, and people they have talked to” [47], it might be suggested that the research displays naturalistic generalisability, and thus, mutual regard for the work.

#### Significant contribution

Given that there are to date, no empirical accounts of the impact of COVID-19 on youth sport in the available literature, the research will clearly make a significant contribution to the field theoretically, heuristically and practically. This should not only be gauged by academic metrics such as scholarly citations in the future, but in the way that this research makes sense of a complex social phenomenon for scholars, sport practitioners and policy makers. It is anticipated that this research will be significant not only because it comprises potentially the first study to explore the impact of COVID-19 on youth sport, but because the research is well designed, rigorously undertaken, and highly accessible to a global audience.

## Results

The reflexive thematic analysis led to the development of four major themes conceptualised as the ‘4 Rs’: *Recognising Struggle; Reconnection; Re-Engaging After Restrictions;* and *Reimagining Sport*. The following results^1^ represent the theoretical work from which the impact of the pandemic on youth sport in South Australia can be understood.

### Recognising struggle

From youth sport participants to senior executives of sporting organisations, a shared experience brought on by the pandemic was emotional *struggle*. The lost sporting season/year for many participants provoked a sense of disappointment and ‘mourning’, particularly because a majority of participants were involved with winter sports that were most affected by the timing of lockdown in Australia. However, the concept of emotional struggle endured when reflecting on the ‘domino effect’ for sporting employees, volunteers and indeed, participants. For instance, the struggles faced by study participants regarding the impact on physical and mental health were perhaps the most profound. Hannah, a 15-year-old netballer, described the constraints placed on her normally active lifestyle by the pandemic’s restrictions: ‘You’re not allowed to go outside, you just sit on the couch, you eat, you sleep, you get – like, we all get a bit lazy through that time’.

While a number of youth participants shared stories about engaging with online fitness programs, home workouts, and general cardiovascular training (long runs, bike rides, swimming) to maintain a level of physical fitness throughout the pandemic, many reported a loss of motivation to continue to keep fit when no end to the restrictions was in sight. Nathan, a 17-year-old footballer, reflected on his engagement with physical activity throughout the lockdown period:

I think my motivation dropped a little bit because you would come home from school, and you’d hear all these rumours of ‘yeah footy is not coming back at all’. I heard that a lot, so I’d just lose a lot of motivation, because I’d be like, ‘oh the season’s done’. I’ll start training again once the season’s back up.

Similarly, Naomi, a mother of a 15-year-old footballer, reflected:

I think, especially with the 15-year-old, he’s going through life changes, he’s got huge amounts of testosterone that he would use up, basically every day doing something footy related, and it was just all gone overnight. It was just, you can’t do this, you’ve got to stop. So, he was managing it okay, and then probably about five weeks in, his mental health started to be affected, became like – I don’t know, I’m not saying aggressive as in physically aggressive, but just the way that he would talk and would just be annoyed at everything, and couldn’t really explain why he was so upset; he became really demotivated for a kid who was very motivated. It just became really hard for him and his mood and mental health went downhill so quickly.

In place of sport training and competition, most youth participants admitted to spending more time on the couch watching TV, eating junk food, and generally experiencing a decline in positive health-related activities. This behavioural change was readily observed by parents and coaches who also expressed concerns about the physical wellbeing of youth sport participants. One father provided an insight into some of the body image-related sensitivities of recognising children’s struggle as a parent.

But, you know, getting back to the sport, she – her, her physical wellbeing has deteriorated. For example, her – she doesn’t – she’s harder to get out of bed to go to school now, whereas before she’d leap out of bed. She’s – I’m not allowed to – this – my daughter’s not going hear this, but she’s put on weight. Right now, my daughter’s a big human being like I am. She’s – the, the exercise has – keeps her in terrific shape, but when she’s not exercising, she – you know, our family are big-boned creatures. That’s what I’ll say. Alright. And, and, you know, and – and that’s such a touchy – I can’t say to my daughter, “you’ve put on weight”, because that is offensive to her, right? But I think the reality is, my wife and I have both said it to each other, Heather’s put on weight, right. So, there’s that actual – I mean, that’s not good for you, you know.

A closely related form of struggle surrounded the impact of cancelled sporting seasons, along with other social sites such as school, on the mental health of youth participants. Parents and coaches perceived more severe mood changes among children, describing symptoms such as becoming easily ‘annoyed’, ‘frustrated’, ‘demotivated’, ‘upset’ and ‘angry’. Several participants theorised that the major contributor to the perceived decline in children’s mental health was the absence of social connectedness that sport participation inevitably provided. Julie, a parent and club committee member, saw the cancellation of sport as the removal of an opportunity for children to socialise: ‘They look forward to the social outing on a Saturday with sport or Thursday night at training and they’re just going to have nothing like that anymore’. Similarly, Claire, a retired teacher with ‘decades of experience’ in youth sport, suggested that the missed opportunity to be involved in sport would have implications for both the immediate mental health of junior athletes, and for the development of mental wellbeing in the future:

… with the isolation, the kids are not good at handling that I don’t think, they need to look at their mates, that sense of loneliness, they sort of feel that the physical activity that they don’t get, they certainly are missing out on that. And the fear of the unknown I think that’s the greatest fear you could have, because they don’t really know what’s going to happen. And so, with all that becomes some dark periods, some negative thoughts and so on. And I just think that sport’s a great one at building resilience. You get whopped in a game and then you bounce back and come back again. And that’s so important because sport reflects what life’s all about and so it’s those qualities, the development of those qualities and attributes that we’re missing out on from there.

This form of emotional struggle was exacerbated during the weeks when schools were shut down during the height of the pandemic in South Australia. However, some like Grace, a 15-year-old netballer, were able to carefully manage a sense of positivity by remaining socially connected online in order to protect mental health throughout the pandemic:

I think without training with my school and without being at school with my friends, it’s kind of put a bit of a downside to my mental stability, but just like being positive each day and just thinking, ‘well the pandemic is not going to last forever and I will see people again’, like it’s definitely something that kept me going. And just like messaging people, FaceTiming, that’s something I did a lot in the pandemic just to keep in touch with the people I enjoy being around.

Struggle was further conceptualised from discussions about the shared disappointment and ‘grief’ stemming from a ‘lost’ sporting season. Tim, an Australian rules football club president, perceived that ‘there would definitely be some junior players who would be grieving that they’ve missed out on their opportunity to play under 16s or even under 18s’. This theme was particularly significant for high performing youth athletes who were on the verge of being, or had been, selected in state and national teams. For them, the emotional struggle pertained to the lost opportunity to compete at national and international events. For talented youth athletes such as Hunter, 15, a sense of disappointment was felt when considering the pandemic’s impact on a potential future pathway into elite sport:

It’s a tough pill to swallow … I’m quite a good footballer and under 15 s is kind of where things get serious. So, when scouts and stuff come down to look, I don’t even know if I’m going to get an opportunity to show them what I can do.

Adding to the complexity of the theme, *struggle,* were organisational stressors that concerned financial loss, redundancies, and concerns about the long-term viability of the sporting club or organisation. At least three participants involved with state-level sporting organisations shared difficult stories about ‘standing down’ staff indefinitely. They grappled with the reality that many of their peers would not return to their organisation. Feelings of guilt and anxiety were only exacerbated when anticipating the broader ramifications for sport program provision to the wider community, which were contingent upon a full season of income and a fully available workforce. John, the CEO of a semi-professional Australian rules football club, reported that all of his staff had been stood down on JobKeeper^1^ payments as a result of the pandemic. Moreover, John’s club had ‘lost’ some elite athletes who were not able to continue to play without match payments if, and when, sport resumed.

### Reconnection

The notion of *reconnection* was a prominent theme among participants, especially when discussing some of the unexpected ‘silver linings’ during the height of the pandemic. Reconnection was philosophically rooted in participants experiences of adapting to life in lockdown with their immediate family, especially in relation to remaining engaged in physical activity. For instance, a sense of reconnected ‘solidarity’ and ‘strength’ was perceived inside the family unit when youth athletes were joined by parents in training activities or general physical activity during lockdown. A powerful example of parental support during lockdown was reported by Samuel, a junior cyclist, whose father purchased a motorbike in order to help pace his son through training rides without access to the regular training squad. Similarly, Maddie, 16, reflected on the support provided by her mother in finding motivation to stay fit during the pandemic:

I definitely drew on my mum because she’s a very fit and active person. She goes to the gym a lot [pre-COVID-19], so just her, she’s just always so empowering. She’ll always push me, and my parents are always there to push me, but just having her home as well. You know, just saying ‘You need to do it’ and ‘You’ll thank me later’, just telling me just to get up and do it … I feel like if I didn’t have her then my fitness levels would have been a lot lower than they were.

Familial reconnection also developed from participants expressing appreciation for, and acknowledging, unique forms of support to provide a sense of normality and regularity during a turbulent and uncertain time. Junior netballer Hannah appreciated the efforts of her parents to provide a sense of normality that encouraged ongoing engagement with healthy habits:

My parents were really keen on making sure that I didn’t, especially with my diet, I guess, sitting at home all the time, it was like eating just for the sake of eating, and that was a big thing. My parents were very helpful with, no, we’re going to make sure we keep the same diet and the same structure of everything. So, like going to bed on time and just that kind of thing.

Parents added an additional perspective to the theme *reconnection*. The social distancing restrictions during lockdown resulted in many children and parents staying home unless parents were deemed an ‘essential worker’ (for example, health professionals and supermarket staff). This period was regularly described by participants, especially parents and coaches, as a time to ‘unplug’ and reconnect as a family unit. Luke, a parent of junior athletes, reported that his wife had been appreciative of the positive impacts the restrictions had on their family: ‘[My wife] thinks the Coronavirus is the best thing that’s ever happened, because the family is spending more time together’. Others shared personal reflections about ‘rethinking’ how much time the family devoted to club sport participation before the pandemic. Many parents felt that the disruption to the weekly routine, which for many families normally involved training sessions and meetings every night of the week, provided a chance for sport-focussed families to spend increased time together during the week. In many cases, the social support missing from sport participation was supplemented by the family.

At a broader level, a sense of connectedness within sporting teams was established and maintained throughout the lockdown period for many youth participants and coaches via online communications. Some participants experienced delayed opportunities owing to the availability of fewer resources and limited communication from their associated sporting club. However, the opportunity to reconnect with coaches and teammates during the pandemic via ‘socials’ (social media platforms) was perceived as vitally important in place of face-to-face interactions. Shannon, a 15-year-old swimmer, provided insights about the meaning of staying socially connected with their peers during the pandemic:

Well with my friends and everything we are quite tight, and we have a good relationship, but I think it [‘Zooming’] was in initiated because of when nationals was cancelled and so many people were disappointed and we were just trying to find the good out of what had happened, and not see all the negatives even though we had to go through that time of grieving, I think we were just all there for each other. Um … I had communication with my coach and our like club committee and everything were posting things or saying stuff to like keep us – like stay positive and everything, and I stayed in contact with a lot of the swimmers, and were all trying to keep each other positive. Like trying to have a positive attitude towards things and where they were going and everything.

At the same time, reconnecting via Zoom™ and FaceTime™ was not always an antidote for an overarching desire to reconnect with the wider sporting community, especially among older family members (typically grandparents) and volunteers who did not have digital literacy skills to connect online and thus relied on community sport to combat feelings of loneliness and seclusion. One participant empathised about the lost social opportunities, particularly for older adults involved with junior sport at present and in the immediate future:

Even with the grandparents, you know, grandpa and grandma always park on the fence at the footy, and 2 cars down it’s Bill and Mary, and they bring their sandwiches and they have lunch together. That meeting, that interaction and all that feel good stuff... You drive past footy ovals and they’re empty, you drive past netball courts and they’re empty … Last night was the first time that I’d been to the stadium, I’d been lots of times and the lights are all off, it’s black and it’s empty, and it gives you that very hollow feeling.

### Re-engaging after restrictions

The theme *re-engaging after restrictions* developed during two significant time periods in South Australia amid the pandemic including: (1) the height of the first ‘wave’ of COVID-19 infections which led to the indefinite postponement or cancellation of sport across South Australia and enforced social distancing restrictions, and (2) the end of the first ‘wave’ of infections as the number of active cases ‘flattened’ to zero, leading to a gradual relaxing of government restrictions that enabled some outdoor competitive sports to resume training in small groups (of less than 10 people). *Re-engaging after restrictions* thus emphasises two distinct notions including the difficulties associated with resuming sport involvement and the participants’ experience of re-engaging with a highly regulated form of sport participation. With respect to returning to sport, several junior athletes reported that they had experienced a lack of motivation to stay fit during the pandemic and struggled to find the drive to continue practising or training. While this represents one avenue through which we explore the difficulties of re-engaging after restrictions, a second perspective concerns those junior athletes who do not return to sport at all in the wake of the pandemic. Re-engaging these youth participants and volunteers comprised a major component of the theme and represents a major challenge for sport administrations and coaches.

Some youth athletes expressed a strong desire (and in some cases, a ‘hunger’) to return to sport, stating that their passion for playing the sport they loved had been strengthened through its absence during lockdown. For this group of junior athletes, re-engaging in sport after restrictions appears a straightforward endeavour. However, the struggle to maintain fitness and skill reported by other junior participants appears to have led to a decrease in confidence and self-efficacy. In some cases, junior participants could ‘see themselves as not being good enough’ to return to sport (as perceived by Claire, retired teacher) because of the disengagement with regular training opportunities. Joanne, a parent of junior sport participants, claimed that it was important to support young people at risk of disengaging from sport by empathising with the range of emotions they may be experiencing: ‘We need to remember to not place pressure on people to return if they don’t feel that that’s something that they are comfortable to do’. Evidently, the ‘key’ to re-engaging the disengaged in the eyes of parents and coaches was to provide opportunities and emotional support for ‘if and when’ youth wanted to return to sport. Following this thematic trajectory, Rebecca, a 15-year-old, reported that some of her peers were experiencing difficulties with re-engaging in sport post lockdown, which was characterised by a series of new COVID-19 restrictions including contact tracing protocols, restricted times for training and restricted group sizes:

I know a lot of people that have dropped out just because they can’t be bothered to continue with the season because it’s too hard to get back into or it’s too hard to follow the restrictions … I know a few people that just don’t want to continue because it’s – I think they don’t find a point.

Where youth were concerned, as opposed to young children 6–10 years, some parents and coaches emphasised the importance of re-engaging with sport after restrictions without being forced. Luke, a father of two junior participants, saw the importance of his children being self-motivated to return to sport rather than being pushed by a parent and was cognisant of his potential influence on his daughter’s return to sport following the easing of restrictions:

My daughter is absolutely no certainty return to sport. In fact, I kind of get the impression she’ll be returning to sport because of my desire for her to do so. Right? And that is not great, I don’t think. As soon as you have to be the one lighting the fire, you might as well stop – well, I don’t want her to stop, but …

The theme re-engagement was also entangled in challenges pertaining to the actual playing experiences post-lockdown which engendered various restrictions and protocols. For some participants such as Hunter, a 15-year-old footballer, the different stages of restriction easing had created momentum and excitement towards a return to ‘normal’:

Last week or the week before, we had to have maximum group of 10. And before training, you have to wash your hands and hand sanitise, clean the balls and do all that sanitation stuff, and then we can go about training, but that was non-contact. So basically, we could just handball, kick and just do lots of running. But now that training’s back, we’ve been able to do a bit – now that we can have 20 people and there’s contact involved. We still have to do the same sanitation, but we can get our ‘hands dirty’, practice some tackling and stuff, which is taking steps in the right direction if we are to have a season.

Conversely, COVID-19 related restrictions had been a frustrating experience for some junior players. Maddie, a junior netballer, reported finding the restrictions in her sport confusing: ‘If you’re in a huddle, we have to stand apart, but then on court, you can get rough against each other, so that’s confusing to me’. Parents also reported frustration at the gathering size restrictions on their children’s participation in sport. Angela, a parent and volunteer, observed that the limits on training numbers may disproportionately affect the junior players who are not among the most highly rated in their age group, and ultimately discourage their continued participation:

I was told that the kids can only train in groups of ten or whatever, but an under-13 age group, they’ve got more than the 30 kids. So, some kids have just been put on an emergency list. To me, I think that’s rubbish. I think that if you’ve got 50 kids in the under-13’s, you find a way to have another training session where the other 20 kids get to have a go. All kids should be able to play sport, and if these restrictions are going to stop kids being able to play, they’ve got to find a way to let them train too, and not make them feel like they’re less important than the first 30 kids.

Coaches also reported difficulty in re-engaging in sport, particularly surrounding the running of a junior program while adhering to social distancing and other restrictions. Participants spoke of the ‘protocols and hurdles’, and the ‘hoops you now have to jump through’ in order to complete a training session. A sense that sport may have changed ‘for good’ was conveyed by a number of study participants. One experienced coach reflected on the potential for the pandemic to change the way sport is run: ‘I’ve had 20 years of coaching footy, and just the freedom [to] do as you please … You can’t do that anymore. It’s not like that.’ However, there remained a strong chorus of concern about the additional layers of administration and organisation imposed on coaches in a ‘post-COVID^2^ world’ which had ‘real potential’ to discourage parents and other volunteers from fulfilling vital coaching and other volunteer roles in the future.

### Re-imagining sport

During the focus groups and interviews, participants regularly reflected on ‘what matters most’ for youth sport and described the pandemic as an opportunity to refocus and *re-imagine sport* once the pandemic recedes. For instance, a common notion conveyed by participants was the re-evaluation of their purpose, responsibility and involvement in sport. John, the CEO of a semi-professional Australian rules football club, faces a post-lockdown season without match payments for players in the elite-level competition. For him, a possible positive to arise from the pandemic is that those who return to that level of sport are ‘there for the right reasons’ and are playing because of their passion for the sport rather than for any financial incentives. John believed that the football program may be stronger as a result of retaining those most passionate: ‘They are doing it for the love of the game … that can be a pretty powerful thing with your program moving forward too, so you feel like you have got people for the right reasons’.

Related to this was the belief of many participants that sport had become too focussed on competition and winning before the lockdown occurred. Alice, who coaches junior netball, observed that children as young as 8 were being placed in ‘A’ and ‘B’ teams, which established a ‘food chain’ and placed unwarranted emphasis on hierarchies of skill and competence over fun and enjoyment. For many participants, the forced period away from sport allowed them to recalibrate toward a deeper and more meaningful connection to sport, underpinned by a profound sense of enjoyment and fun that they first experienced when participating. Maddie, 16, believed that sport after lockdown should place more emphasis on fun:

I would like it to be more enjoyment rather than, I guess … success or any of that. I think it needs to start with enjoyment. If everyone sort of comes in with a mindset of being the best or winning premierships or any of that kind of stuff, it kind of takes away from what sport in in our Australian culture. I think the social aspect of sport, it’s such an important [thing].

Other participants mentioned the many benefits that sport brings by way of a sense of social connection and community. Claire, 82, stated that a shift in focus from the actual playing of sport to the many wider benefits that sport brings would occur as participants reflect post-lockdown:

I don’t think many people realise just how important sport was, it’s not just kicking goals or throwing goals or, you know, taking marks or placing tackles or dribbling up the court, it doesn’t matter. That’s simply the vehicle for the wellbeing of communities and that social fabric.

As participants considered how they would like sport to be seen in a ‘post-COVID world’, many participants claimed that the benefits to physical and mental health should be more forcefully valued and highlighted by sporting clubs and communities. Rose, 15, suggested that efforts to re-engage people with sport should centre around a message of enjoyment and positivity:

I would like to see a message that makes the sport appear really fun and enjoyable, and very social, so you’re not just trapped in four walls, that you’re being outside, you’re having fun and with people that you really connect with … it could motivate them to join a social club and play a sport, or just go out for a run and that sort of thing.

In addition to re-imagining the philosophical architecture of youth sport participation, coaches and administrators of youth sport shared some bespoke ideas about how sport might need to be rebranded in the future. This was driven in part by a concern about how the pandemic might discourage parental and volunteer efforts in the future, particularly those who have significant health related risks. Similarly, several participants acknowledged that youth sport might need to be reimagined because the forced hiatus from sport caused by the pandemic may prompt families to reassess the time commitments devoted to children’s sport. For Angus, who works in community engagement for a sporting organisation, the provision of more flexible family participation (‘flexi-time’) may be one novel way in which sports evolve in the future:

People have seen what it’s like to not have all the choices to be able to do what they want to do, and I think they’re going to choose their time a bit more carefully now. It’s one of those legacy things I think out of COVID-19 that it’s really opened up people’s eyes as to what happens when you don’t have something that you can just opt into.

Other concerns shared by coaches and administrators were around using the forced time away from sport to make changes that had been discussed for years prior to the pandemic, but never actioned. For these participants, there was a feeling that the lockdown period should be a catalyst for promoting overdue change. For instance, Sally, an athletics administrator, believed that her sport should return with more of a focus on flexibility of choice, so that ‘kids can actually do the events that they like and don’t have to do the events that they don’t like’. The meaning of reimagining sport was thus a significant theme through which the pandemic forced youth sport stakeholders to reflect on and conceptualise new ideas for the future. Some ideas involved ‘getting back to basics’ by prioritising fun experiences, while others involved innovative and novel adjustments to help youth sport navigate their way forward after the pandemic. Others mentioned that they were currently developing new ways to revitalise their sporting communities by developing dedicated YouTube channels and podcasts to share information and prepare for the next potential forced hiatus from sport.

## Discussion

The objective of this study was to explore the perceived impact of COVID-19 on youth sport from a range of stakeholders. Two key research questions guided the study: (1) What impact has the COVID-19 pandemic had on youth sport? and (2) How has the COVID-19 pandemic influenced attitudes and behaviours surrounding future youth sport participation? The findings indicate substantial and complex consequences broadly conceptualised as the ‘4 Rs’, encapsulating the *recognition* of emotional struggle, the *reconnection* of family units and social networks, the complex issues surrounding the *re-engagement* of sport participants and volunteers, and the careful *re-imagining* of the purpose and meaning of youth sport moving forward for families and communities. Given the dearth of empirical literature surrounding the youth sport setting within the context of the global pandemic, we offer a timely focus on the implications of this research for sporting clubs, organisations and families to navigate a future return to sport.

The rapid decline in mental health brought about largely from social isolation represented a major form of emotional struggle for families and youth athletes specifically. Other forms of struggle surrounded a lack of motivation for individualised training and general physical activity. The identification of the emotional struggle youth, parents and families more broadly endured during the pandemic prompts sporting clubs and organisations to consider their broader role and responsibility in youth sport. Youth sporting clubs have been previously identified as opportunistic settings for mental health interventions [9] and social support in general (for review, see [48]). However, the provision of social and emotional support in youth sport has been almost exclusively dependent on access to the sporting club. In contrast, the forced hiatus from sport as a result of the pandemic removed the structural elements that fostered strong social support and the delivery of mental health programs. In this regard, sporting clubs and sporting leaders are encumbered to reflect on and consider their role in facilitating continued social and emotional support, particularly under conditions such as that of a global pandemic or other catastrophes (e.g. bushfires, global recessions) which force an indefinite closure of or limit access to youth sport.

For the proactive sporting club determined to support their stakeholders, the creation and sharing of resources could be one avenue worthwhile exploring. Enhanced platforms for podcasting, video blogging and conference calling may be useful in supporting for youth and families during times of significant and unprecedented disruption to sport. By enhancing skills and resources, a youth sport club or organisation may be better positioned to help alleviate uncertainty, provide mental health first aid, and promote ideas for active sport engagement in the home environment (e.g. individualised training sessions), all of which may assist stakeholders cope during unprecedented times.

In situations where sporting clubs are well-resourced, clubs might benefit from drawing on evidence-based ideas that optimise the development of videos and other digital resources for parents and families. For instance, evidence-based ideas for the development of targeted video materials have been proposed recently in relation to developing educational support resources for parents in youth sport [see 50]. Though decontextualized from the recent pandemic, Kwon and colleagues [49] provide a point of departure for sporting clubs and organisations to improve communication strategies and resource creation for youth sport stakeholders.

For others who do not have the resources but wish to be prepared for ‘next time’, the obvious barrier includes a lack of capital, in terms of intellectual and human resources. Consequently, another strategy might involve partnering with researchers and universities for competitive funding to enable the development of resources and training programs designed to help sporting clubs support families during unprecedented times. In Australia, competitive research schemes promote national and international partnerships between researchers and business, industry, and community organisations in order to apply advanced knowledge to problems. These funding opportunities foster innovative thinking that may assist clubs and sporting organisations to play a meaningful role in helping youth sport stakeholders manage the emotional struggles that appear and endure during unexpected catastrophe. Alternatively, a low-cost solution might involve a stronger emphasis on establishing community partnerships (e.g. between rival sporting clubs, or between sporting clubs and schools) to combine intellectual and material resources to address key challenges, promote the exchange of ideas, and explore innovative ways to rebuild the volunteer workforce. A cohort of physical education students from a local high school or sport management undergraduates are just two examples whereby community sport may benefit from developing intersectoral partnerships with the wider community, especially in the wake of COVID-19.

Although the incidence of domestic violence and abuse have purported risen during the global pandemic [50], the present findings situated within the largely homogenous context of youth sport in South Australia indicate that familial relationships strengthened during the pandemic because of increased time spent in the domestic setting. However, this did not always translate into a positive influence on children’s sport related behaviour during the pandemic as many youth athletes perceived a decline in their overall fitness and sport motivation. This raises questions about *all* parents’ capacity to support and maintain children’s motivation, interest in and connection to sport during unprecedented conditions such as that of a global pandemic. In the spirit of being better prepared for the next unexpected disruption to sport, it is important to acknowledge that some parents do not necessarily possess the confidence, knowledge, skills and/or resources to ultimately maintain their children’s sense of ‘connection’ to sport. To elucidate this perspective, previous research has indicated that many parents are uncertain how to engage in youth sport and how best to support their child [51]. Consequently, sporting clubs and organisations may seek to better understand the nature of sport parenting during the pandemic to target future intervention and support efforts to optimise parents’ influence on children’s sporting involvement from the home setting. Parents may benefit from access to advice, training resources and ideas to help them step into the role of ‘home coach’ or ‘encourager’. They may also benefit from tips and informational support to encourage and reinforce their children’s motivation for, and connection with sport. Sporting clubs could share resources across the sector to support families in this regard and/or embark on the creation and production of their own tailored materials.

The findings also prompt sporting clubs and organisations to think about how families and volunteers will be re-engaged after restrictions. At present, the return to sport experience is anticipated to be highly regulated to ensure that clubs and participants comply with strict COVID-policies concerning participation. However, this may impact the overall enjoyment and experience of sport for young people, especially those who prefer a version of sport akin to the “pre-COVID” sporting period. For instance, the policing of ‘high fives’, hugs and other forms of physical embrace seemingly have the potential to diminish a key aspect of what youth, parents and sport providers consider ‘fun’ in sport [10, 52, 53]. More broadly, clubs will need to innovate new ways to entice families including volunteers back to sport. While many will naturally return with enthusiasm and energy, other families may be less inclined for a variety of reasons. For instance, sporting clubs and teams will need to be sensitive to the possibility that some families may not be able to financially enable children’s sporting opportunities. They will also need to acknowledge that families may decide to reduce their commitments to fewer sporting activities [30]. Consequently, new methods for attracting and retaining volunteers and participants will be key. Although it is beyond this study to provide evidence-based recommendations in this regard, future research could investigate how clubs are seeking to re-engage families as a means for developing further strategies in a ‘post-COVID’ context. Some strategies might begin with furthering efforts to develop quality coaching practices by working with ‘master coaches’ (coach developers) [54] to enhance youth sport experiences. Alternatively, the recent surge in the uptake by coaches surrounding online learning and development opportunities during the pandemic [55] might result in improved sporting experiences.

In addition to coaches, sporting clubs at large may need to find ways to attract young people’s interest and time. One strategy might involve a consideration of Elliott, Bevan and Litchfield’s [56] grounded theory for attracting youth sport participants. Although the grounded theory was derived from focus groups with female youth footballers, the theory provides an important foundation for sporting clubs to inform decisions about re-engaging young people and volunteers into sport. For instance, understanding Elliott and colleagues’ theorisation of the concept ‘identity’ may prompt sporting clubs to reflect on how they can ‘connect’ influential sporting figures with youth sport participants via stories that stimulate sporting interest to create a sense of identity with future participants.

Similarly, clubs may wish to refresh their understandings and practices that align with the concepts of fun and enjoyment. Recent research suggests that clubs and organisations do not always optimise fun experiences in sport [10], offering a potential opportunity for sporting clubs and organisations to enhance the range of internal, external, social and contextual factors that create a fun and enjoyable sporting experience. This cannot be underestimated given the largest peak for youth sport drop-out occurs during adolescence, which may increase because of COVID-19. These points of departure are timely and highly relevant at a time when families and volunteers may require assistance re-engaging in sport [30].

A final implication for sporting clubs and organisations is to consider how many youth sport stakeholders are in a process of reimagining sport for the future. The most recent priorities for youth sport prior to the pandemic implored researchers and practitioners to focus on improving participation and retention, promoting greater accessibility for participants, addressing the complex relational challenges between parents, coaches, administrators and athletes, and maximising enjoyment [25, 26]. These focus areas are likely to assume added significance as sporting clubs and organisations navigate the slippery and uncertain times ahead. However, additional priorities may also emerge for the field, reflecting the nature of youth sport at present, particularly in relation to re-envisioning the purpose and meaning of youth sport participation. For instance, a recalibration of expectations about training may develop from strict training protocols that reduce the number of players working in a designated playing space. Further, clubs may seek to revise their ‘brand’ following the pandemic, from merely a place of sport involvement toward a multifaceted community of practice in which sport is a vehicle for socialisation. The social nature of community sport could be ‘branded’ as improving social, mental, and physical health, and with a ‘new’ focus on how fun and enjoyment and the competitive nature of sport can co-exist as part of a holistic sporting endeavour. In this way, sporting clubs serve a more comprehensive purpose for youth sport stakeholders and the wider community.

To our knowledge, this is the first study to explore the impact of the pandemic on youth sport. The findings, conceptualised as the ‘4 Rs’, provide rich, detailed insights into the perceptions and experiences of how youth sport has been impacted by COVID-19. However, a potential limitation of the study is the sampling bias regarding the participants interviewed so it is possible that those with the time and interest in participating in this study are potentially not those most affected by COVID-19. Parents and families that are highly educated and already supportive of their children’s sporting interests may be more likely to self-select into such a study, meaning that the voices represented in this paper may not be truly representative of the struggle faced by those involved with youth sport during COVID-19. The severity of the impact may not yet be fully realised, which is something that researchers need to consider when working with youth sport stakeholders into the future.

## Conclusions

COVID-19 and the absence of community sport has dramatically highlighted the core values of community sport. That is, social connectedness through fun and engaging play. From this individual participants, families, volunteers, and communities have many benefits including social, mental and physical health. We now need community sport to continue with these core values to revive youth sport participation and retention. Communication and connecting with players and volunteers are going to be key for sports success once the pandemic recedes. Additional resources and tools are also necessary to ensure that sport, both in Australia and globally, can rebound from the pandemic. We hope the ‘4 Rs’ offer sport providers and researchers with a theoretically-informed point of departure for pursuing these avenues.

## Supplementary Information

**Additional file 1. File 1:** Interview guide (adults). Semi-structured interview guide

**Additional file 2. File 2:** Focus group guide/interview guide (children). Semi-structured interview guide

## Data Availability

The datasets generated during and/or analysed during the current study are not publicly available due to them containing information that could compromise research participant privacy/consent but are available in a de-identified format from the corresponding author on reasonable request.

## Abbreviations

COVID-19: Abbreviation for the novel coronavirus SARS-CoV2
LGBTIQ+: Acronym for Lesbian, Gay, Bisexual, Trans, Intersex and Queer
BMX: Acronym for Bicycle motocross

## Footnotes

1 Any names referenced in the quotes within the results are pseudonyms.

2 The term ‘post-COVID’ in the context of these results refers to the period following the height of the pandemic in South Australia when restrictions were relaxed, allowing some sports to return to training under adapted, restricted conditions.
