# Supplementary material for: Understanding the impact of COVID-19 on youth sport in Australia and consequences for future participation and retention
Source: BMC Public Health. 2021 Mar 5;21:448. doi: 10.1186/s12889-021-10505-5 (PMC7935002; doi:10.1186/s12889-021-10505-5)
Supplement: Supplementary file 1 — Additional file 1. File 1: Interview guide (adults). Semi-structured interview guide [file 12889_2021_10505_MOESM1_ESM.docx]

**Semi-structured interview guide (adults)**

- How has the COVID-19 pandemic impacted your sporting organisation/club?
- What opportunities (if any) do you perceive might come out of the Pandemic for your sporting club/organisation?
- What do you perceive will be the short, medium and long-term challenges for your sporting/club organisation?
- How can other sectors assist sporting organisations/clubs to get back on their feet?
- A number of youths have described the ‘lost season’ as ‘heart breaking’, many who may not return to sport as a result. With this in mind, how would you like sport to be marketed on the other side of COVID-19?
- (For parents) - How are your children coping at the moment without sport?
- Others suggest that there could be a massive surge in sport demand, well beyond what the industry can service. What are the pressure points you anticipate after COVID-19 recedes for your club to manage participation?
- Community sport relies on volunteers, many of which are working parents. How will clubs work with parents and volunteers to juggle time demands between working more and supporting sport? What plans is your club putting in place to assist?
- If there is a surge in participation demand, what has your club/organisation put in place to accommodate increasing participation?
- If there is a sharp decline in youth sport participation, what strategies has your club/organisation developed to manage the fallout?
- In what ways will your sporting club/organisation operate differently (if at all) post Pandemic?
- Had you ever planned previously that something like this might occur?
- How might you now view the future in terms of how you operate when this is all over?
- What do you foresee will be the future of community based sport in the wake of COVID-19?
