# Supplementary material for: Understanding the impact of COVID-19 on youth sport in Australia and consequences for future participation and retention
Source: BMC Public Health. 2021 Mar 5;21:448. doi: 10.1186/s12889-021-10505-5 (PMC7935002; doi:10.1186/s12889-021-10505-5)
Supplement: Supplementary file 2 — Additional file 2. File 2: Focus group guide/interview guide (children). Semi-structured interview guide [file 12889_2021_10505_MOESM2_ESM.docx]

**Semi-structured interview/focus group guide (children)**

- To begin with and make sure our audio is working could I please ask for your names, your age and maybe what sport you play?
- In your own words how has Covid-19 impacted your sport this season?
- How has the pandemic impacted your attitude and motivation towards sport? What about your peers?
- How have you spent your time during the pandemic that would otherwise be spent at training and games?
- Tell me a little bit about how your family have adopted to the cancellation of sport this season.
- What has been the nature of communication from coaches and your sporting clubs during the pandemic?
- How would you describe your overall mental health and wellbeing in the context of a ‘lost’ sporting season?
- When sport returns, how would you like sport re-engage people to come back to sport?
- What do you perceive will be the short, medium and long-term challenges for your sporting/club organisation?
- How can other sectors assist sporting organisations/clubs to get back on their feet?
- In what ways do you think your sporting club/organisation might do things differently (if at all) post Pandemic?
- What do you foresee will be the future of community based sport in the wake of COVID-19?
